# Impact of adding carboplatin to docetaxel chemotherapy on testosterone levels and treatment outcomes in metastatic docetaxel-resistant prostate cancer

**DOI:** 10.1038/s41598-025-04667-0

**Published:** 2025-06-20

**Authors:** Hejar Atalan, Michael A. Morgan, Philipp Ivanyi, Paula Kappler, Florian H. Heidel, Christoph W. M. Reuter

**Affiliations:** 1 Department of Hematology, Hemostasis, Oncology and Stem Cell Transplantation, Hannover Medical School, Carl-Neuberg-Str. 1, 30625 Hannover, Germany; 2 Institute of Experimental Hematology, Hannover Medical School, Carl- Neuberg-Str. 1, 30625 Hannover, Germany

**Keywords:** Prostate cancer, Docetaxel resistance, Carboplatin, Testosterone, Medical research, Oncology, Urology

## Abstract

Docetaxel resistance, particularly post-androgen-receptor targeted therapy (ART), undermines its clinical utility in metastatic castration-resistant prostate cancer (mCRPC). This study explores the impact of docetaxel plus carboplatin (DC) chemotherapy on serum testosterone levels in metastatic docetaxel-resistant prostate cancer (mDRPC) patients. 123 mDRPC patients were categorized into three groups: (1) no previous ART (*n* = 65), (2) previous ART with serum free testosterone (FT) > detection limit (DL) at baseline (*n* = 31), and (3) previous ART with FT < DL at baseline (*n* = 27). Salvage DC chemotherapy led to significant reductions in FT and total testosterone (TT) levels in groups 1 and 2 (*p* < 0.05). Group 1 saw FT decrease from 0.85 pg/mL to below the DL (< 0.18 pg/mL) with 54.3% achieving complete reduction (CR); group 2 showed FT decrease from 0.28 pg/mL to below the DL with 67.7% achieving CR; group 3 had baseline FT values already below the DL with 96.3% maintaining this level. TT reductions to below the DL occurred in all groups. Low FT was an independent predictor for better PFS and for improved OS in groups 1 and 2. Our data indicate that adding carboplatin may improve the therapeutic effects of docetaxel in a castration-dependent setting.

**Supplementary Information:**

The online version contains supplementary material available at 10.1038/s41598-025-04667-0.

## Introduction

The pathophysiology of prostate cancer underscores the significance of androgen receptor (AR) activation and AR-driven transcriptional programs, making androgen deprivation therapy (ADT) and androgen-receptor targeted therapy (ART) essential treatments^1^. However, resistance inevitably emerges, leaving metastatic prostate cancer incurable^1^, often due to the reactivation of AR pathways despite suppressed testosterone levels^2^.

Docetaxel chemotherapy presents as a promising option, providing an excellent initial response and considerable survival benefits in metastatic castration-naive (mCNPC) and castration-resistant prostate cancer (mCRPC) patients^3^. However, its clinical utility is compromised when primary and acquired resistance occur^3^, with the definition of docetaxel resistance still debated^4^. It has been suggested that docetaxel resistance should be considered evidence of disease progression if it occurs during therapy or within 30–60 days after the last drug administration^4,5^.

Apart from cabazitaxel and in the absence of alternative effective chemotherapies, docetaxel rechallenge remains a possible option for patients with proven sensitivity to docetaxel^6^.

In a retrospective study, 50 out of 148 patients who initially responded to first-line docetaxel received docetaxel rechallenge. Twenty-four patients (48%) experienced a 50% decrease in prostate-specific antigen (PSA) levels (95% CI 34.1–61.8%). The median overall survival (OS) was 16 months (95% CI 13–20)^7^. Only two randomized trials (GETUG-AFU 15, RECARDO) have reported the outcomes of docetaxel rechallenge. In the RECARDO trial, docetaxel alone or in combination with carboplatin were tested in mCRPC patients who had progressed after responding to prior docetaxel treatment. The study, which was discontinued early due to insufficient recruitment, reported a comparable outcome: a PSA response (PSAR) rate of 35.1% vs. 42.1%, a progression-free survival (PFS) of 12.7 months (95% CI 9.9–17.5) vs. 11.7 months (95% CI 8.5–21.0), and an OS of 18.5 months (95% CI 11.8–24.5) vs. 18.9 months (95% CI 16.0-23.7)^8^. The GETUG-AFU 15 trial investigated the efficacy of doce-taxel rechallenge as a salvage therapy in mCRPC patients previously treated with upfront ADT plus docetaxel for mCNPC. The outcome was disappointing: In this population, docetaxel rechallenge resulted in a PSAR rate of 20% (95% CI 6–44) and a biochemical PFS of 3.4 months (95% CI 0.9–6.8), indicating limited efficacy^9^.

Notably, docetaxel treatment substantially reduces androgen levels, including total testosterone (TT), leading to improved OS^10^. Furthermore, serum total and free testosterone (FT) level reductions during docetaxel chemotherapy correlate with better PFS and OS, particularly in patients without a history of ART^5^. Interestingly, FT emerges as a considerable predictor for treatment outcomes, underscoring its biological importance in the context of mCRPC therapy^5^.

Docetaxel was previously reported to be less efficient after treatment with ART, with a reduced PSAR rate after ART (median PSAR group 1: docetaxel → cabazitaxel → ART: 59.8%; group 2: docetaxel → ART → cabazitaxel: 64.3%; group 3: ART → docetaxel → cabazitaxel: 44.0%; *p* = 0.021) and significantly shorter PFS and OS (median radiographic PFS group 1: 26.9 (range 14.8-not reached) months, group 2: 11.0 (range 9.5–12.9) months, group 3: 6.6 (range 5.0-10.2) months, *p* < 0.001; median OS group 1: 34.8 (range 32.4–41.5) months, group 2: 35.8 (range 33.9–38.4) months, group 3: 28.9 (range 23.3–35.9) months, *p* = 0.007)^11^. A possible cross-resistance between docetaxel and ART was discussed as a potential explanation for these findings^11^.

A recent systematic review and Bayesian network analysis evaluating second-line treatment options in 5,388 mCRPC patients after progression on first-line ART showed that second-line docetaxel resulted in a PSAR rate of 40% (range 24.0-47.8%), a PSA-PFS of 5.3 (range 4.1–7.7) months, a radiographic PFS of 7.5 (range 4.4–9.8) months and an OS of 16.9 (range 10.5–19.5) months^12^. However, in the first-line setting, a median PSAR rate of 56.4% (range 41.0-67.3%) and a median OS of 18.9 (range 15.3–21.7) months were reported for docetaxel treatment of mCRPC patients in an analysis of 13 trials^13^.

Platinum-taxane combinations have demonstrated substantial clinical efficacy in mCRPC^14–16^. Of note, platinum agents (e.g., cisplatin) profoundly disrupt normal testosterone production and impact the hypothalamic-pituitary-testis steroidogenesis axis^17^. Within the testes, cisplatin reduces mitochondrial cytochrome P450 and microsomal 17 ɑ-hydroxylase activity and affects Leydig and Sertoli cell functions. The effects on cytochrome P450 extend beyond the testes to other organs, including the liver and kidneys^17,18^. Platinum complexes have been shown to decrease plasma testosterone levels in male rats by inhibiting steroidogenic enzymes, such as cholesterol side chain cleavage enzyme (CYP11A1), 3β-hydroxysteroid dehydrogenase/Δ4Δ5-isomerase (HSD3B1,2) and 17α-hydroxylase/17,20-lyase (CYP17A1)^19–22^. Carboplatin was found to be less toxic on the steroidogenesis function of the Leydig cells^23^, but this effect may have been underestimated in earlier studies because only TT was measured.

To expand on these observations, we examined the effect of docetaxel plus carboplatin (DC) on serum FT and TT levels in metastatic docetaxel-resistant prostate cancer (mDRPC) patients. Our aim was to assess whether the DC combination decreases testosterone levels and overcomes resistance to docetaxel therapy after prior ART treatment.

## Methods

### Patient selection

In this retrospective biomarker translation study, data were collected from patients with histologically confirmed, metastatic castration- and docetaxel-resistant prostate cancer treated at Hannover Medical School. The patients were followed up until death and had previously received a minimum of three cycles of docetaxel (i.e., cumulative dose > 225 mg/m²) to eliminate PSA flare as a potential bias source. Inclusion criteria were resistance to docetaxel and evidence of disease progression during chemotherapy with docetaxel. In this context, disease progression was defined by the Prostate Cancer Working Group (PCWG) 2/3 as (1) two consecutive increases in PSA of ≥ 25% and ≥ 2 µg/L above the nadir on two consecutive measurements at least four weeks apart and/or (2) progression of measurable disease (according to Response Evaluation Criteria in Solid Tumors (RECIST) version 1.1) and/or (3) appearance of two or more new bone lesions^24–26^. The patients were categorized into three groups: (1) mDRPC without previous ART (i.e., abiraterone and/or enzalutamide) administration (*n* = 65), (2) mDRPC with previous administration of ART and serum FT level > detection limit (DL) at baseline (*n* = 31), and (3) mDRPC with previous administration of ART and FT level < DL at baseline (*n* = 27). The study adhered to the Declaration of Helsinki and Good Clinical Practice guidelines, and was approved by the Hannover Medical School institutional review board (13th August 2008). Informed consent was obtained from all patients in accordance with institutional guidelines. Most patients were discussed at the tumor board according to our oncology clinic protocols. This process remained unchanged throughout the 19-year study period.

### Treatment plan

Patients with mDRPC received a minimum of two cycles of carboplatin AUC5 intravenously for 30 min on day 1 every 4 weeks (q4w), docetaxel at a dose of 35 mg/m^2^ intravenously for one hour each week on days 1, 8, 15 plus prednisone 2 × 5 mg a day orally until disease progression or occurrence of intolerable adverse effects. Standard dexamethasone and anti-emetic premedication were used, and ADT was maintained throughout treatment. Accompanying use of bisphosphonates or denosumab was allowed. Granulocyte-colony stimulating factor (G-CSF) was not used routinely.

The pre-treatment evaluations included medical history, physical examination, complete blood cell count, chemistry profile, serum PSA, neuron-specific enolase (NSE), chromogranin A (CgA), FT and TT levels, bone scan and computed tomography (CT). Morning FT and TT (8–11 am) were measured before and preferably weekly during treatment using an enzyme immunoassay (ELISA from IBL, International GmbH, Hamburg, Germany) and a direct, competitive, chemiluminescence immunoassay (LIAISON Testosterone Assay, Diasorin S.p.A., Saluggia, Italy)^5^. The patients were evaluated for response according to the PCWG 2/3 and RECIST version 1.1 recommendations by imaging studies every three cycles (12 weeks) or if tumor progression was suspected^24–26^. PSA levels were measured at least every cycle and post-treatment changes were defined on the basis of the degree of change from baseline. Before and during the therapy, a pain history was conducted through clinician interview. The severity of adverse events was consistently graded in accordance with the Common Terminology Criteria for Adverse Events version 3.0 (CTCAE).

### Data analysis

The primary study endpoint of OS was calculated from the first date of DC treatment to death. Secondary endpoints included PFS, defined as the time between DC treatment initiation and first date of progression according to PCWG 2/3 criteria^24,26^, PSAR, defined as a ≥ 50% decline in PSA from baseline, and serum FT and TT levels at baseline and during treatment. The RECIST version 1.1 criteria served as the standard for measurement of the radiographic response^25^. OS, PFS and PSAR were analyzed with respect to various baseline and treatment-dependent parameters, including age, Eastern Cooperative Oncology Group (ECOG) performance status, Gleason score, primary or secondary failure towards docetaxel, metastatic lesions, number of previous docetaxel-based cycles, previous prednisone treatment, thrombosis, anticoagulation, radiotherapy, pain reduction, hemoglobin (Hb), neutrophil-to-lymphocyte ratio (NLR), serum c-reactive protein (CRP), alkaline phosphatase (AP), lactate dehydrogenase (LDH), NSE, CgA, FT, TT and PSA levels, and PSA flare.

Statistical analyses were conducted at a 95% significance level using IBM SPSS Statistics version 29. Patient characteristics were summarized using median (range) for numeric variables and frequencies (percentages) for categorical variables. Significant differences between the groups at the beginning of the treatment and throughout the course were assessed using t-tests and chi-square tests. In this regard, statistical significance was defined as *p* < 0.05 for all comparisons. Logistic regression was used to analyze the prognostic significance of patient and disease characteristics in predicting a decrease of ≥ 50% in PSA from baseline. Moreover, univariate and multivariate Cox proportional hazards regression modeling was used to assess the prognostic significance of patient and disease characteristics concerning both PFS and OS. The distributions of PFS and OS were estimated by the Kaplan-Meier method. Non-proportionality was assessed through graphical analysis of Kaplan-Meier survival distributions and log(-log(survival probability)) plots for each covariate level, with parallelism indicating proportional hazards^27^. Additionally, to exclude potential lead time bias, extended Cox modelling with time-by-covariates and conditional landmark analyses were conducted^28^.

Effect sizes, hazard ratios, and confidence intervals were reported to demonstrate the robustness of the findings. To account for multiple hypothesis testing, significance adjustment was performed using the Bonferroni correction and the Benjamini-Hochberg formula^29–31^.

## Results

### Patient characteristics

From February 2005 to September 2023, a total of 123 patients with mDRPC received a salvage chemotherapy with DC. The pre-treatment consisted of docetaxel alone (group 1, *n* = 65), docetaxel as well as ART (group 2: FT baseline level > DL, *n* = 31), or docetaxel as well as ART (group 3: FT baseline level < DL, *n* = 27).

The median age at baseline was 69 years. Each group had a median involvement of two metastatic organs. Overall, 96.7% of the patients had bone metastases, while 63.4% had soft tissue metastases.

As demonstrated in Table 1, baseline demographics, patient and disease characteristics differed between the groups. Patients in group 2 had a significantly shorter duration of ADT and exhibited significantly more primary resistance to docetaxel (61.3% vs. 43.1% in group 1 and 25.9% in group 3). Group 2 patients were significantly more likely to have soft tissue metastases only and less likely to experience PSA progression only. Their docetaxel chemotherapy consisted of significantly fewer cycles (median of 6 vs. 9 in group 1 and 10 in group 3) and was administered for a significantly shorter duration (median of 5 months vs. 7 months in group 1 and 10 months in group 3). Consequently, their cumulative docetaxel dose was significantly lower (median of 450 mg/m² vs. 735 mg/m² in group 1 and 850 mg/m² in group 3).

**Table 1:** Patient characteristics, prior treatments and laboratory values at baseline.

	All (mDRPC) *n* = 123	Group 1 (mDRPC w/o ART) *n* = 65	Group 2 (mDRPC + ART: FT > DL) *n* = 31	Group 3 (mDRPC + ART: FT < DL) *n* = 27	*p* value*
**Patient characteristics**					
Age (years), median (range)	69 (50–80)	68 (52–78)	68 (50–77)*	71 (60–80)*	< 0.05
ECOG, median (range)	1 (0–3)	1 (0–3)	1 (0–3)	1 (0–3)	
Gleason score 8–10, no. (%)	77/116 (66.4)	39/61 (63.9)	23/30 (76.7)	15/25 (60.0)	
Metastases, median (range)	2 (1–4)	2 (1–4)	2 (1–4)	2 (1–4)	
Bone	119 (96.7)	61 (93.8)	31 (100)	27 (100)	
Soft tissue	78 (63.4)	37 (56.9)	23 (74.2)	18 (66.7)	
Lymph nodes	59 (48.0)	27 (41.5)	18 (58.1)	14 (51.9)	
Lungs	23 (18.7)	12 (18.5)	5 (16.1)	6 (22.2)	
Liver	33 (26.8)	16 (24.6)	11 (35.5)	6 (22.2)	
Presence of pain, no. (%)	86 (69.9)	42 (64.6)	26 (83.9)	18 (66.7)	
Non-narcotics required, no. (%)	64 (52.0)*	28 (43.1)*	23 (74.2)*	13 (48.1)*	< 0.05
Narcotics required, no. (%)	54 (43.9)	26 (40.0)	18 (58.1)	10 (37.0)	
Progression before entry, no. (%)					
Bone and soft tissue metastasis	48 (39.0)	24 (36.9)	13 (41.9)	11 (40.7)	
Bone metastasis only	32 (26.0)	20 (30.8)	8 (25.8)	4 (14.8)	
Soft tissue metastasis only	22 (17.9)	8 (12.3)*	9 (29.0)*	5 (18.5)	< 0.05
PSA only	21 (17.1)*	13 (20.0)*	1 (3.2)*	7 (25.9)*	< 0.05
**Prior treatments**					
Local therapy, no. (%)					
Prostatectomy	55 (44.7)	27 (41.5)	15 (48.4)	13 (48.1)	
TURP	22 (17.9)	17 (26.2)*	2 (6.5)*	3 (11.1)	< 0.05
Radiotherapy, no. (%)					
Prostate	59 (48.0)	32 (49.2)	15 (48.4)	12 (44.4)	
Bone	57 (46.3)	28 (43.1)	18 (58.1)	11 (40.7)	
Soft tissue	28 (22.8)	10 (15.4)	9 (29.0)	9 (33.3)	
Chemotherapy					
Regimens, no. (%)					
1	92 (74.8)	48 (73.8)	25 (80.6)	19 (70.4)	
2	27 (22.0)	13 (20.0)	6 (19.4)	8 (29.6)	
3	4 (3.3)	4 (6.2)	0 (0.0)	0 (0.0)	
Characteristics					
No. of docetaxel cycles, median (range)	9 (2-108)	9 (3–44)*	6 (2–26)*	10 (4-108)*	< 0.05
Duration of docetaxel (months), median (range)	7 (2–78)	7 (3–50)	5 (2–32)*	11 (3–78)*	< 0.05
Cumulative docetaxel dose (mg/m²), median (range)	675 (200-8,100)	735 (225-6,600)	450 (200-1,950)*	850 (356-8,100)*	< 0.05
Primary resistant towards docetaxel, no. (%)	54 (43.9)	28 (43.1)	19 (61.3)*	7 (25.9)*	< 0.05
Time (months), median (range)					
From diagnosis of metastatic disease to start of DC	36.9 (6.6-147.5)	34.4 (6.6-140.9)	38.2 (11.6-108.9)	43.3 (13.4-147.5)	
From last docetaxel alone to start of DC	2.9 (0-42.8)*	1.2 (0-19.6)*	5.8 (0-40.5)*	3.6 (0-42.8)*	< 0.05
Radiopharmaceuticals, no. (%)					
Samarium	6 (4.9)	5 (7.7)	0 (0.0)	1 (3.7)	
Alpharadin	5 (4.1)	1 (1.5)	3 (9.7)	1 (3.7)	
PSMA-targeted therapy	2 (1.6)	0 (0.0)	1 (3.2)	1 (3.7)	
ART, no. (%)					
Abiraterone	49 (39.8)*	0 (0.0)*	25 (80.6)*	24 (88.9)*	< 0.05
Enzalutamide	33 (26.8)*	0 (0.0)*	18 (58.1)*	15 (55.6)*	< 0.05
Other therapies					
Duration of prior ADT (months), median (range)	27 (0-123)	26 (2-123)	22 (0-101)*	36 (0-108)*	< 0.05
Estramustine, no. (%)	25 (20.3)*	20 (30.8)*	2 (6.5)*	3 (11.1)*	< 0.05
Ketoconazole, no. (%)	7 (5.7)	4 (6.2)	2 (6.5)	1 (3.7)	
**Laboratory values**					
PSA (µg/L), median (range)	209.4 (9.9–11,928)	210.6 (10.18-11,928)	243.0 (17.0–2,397)	96.3 (9.9-4,397)	
FT (pg/mL), median (range)	0.32 (0.01-16)*	0.85 (0.21-16)*	0.28 (0.02–9.1)	0.01 (0.01–0.18)*	< 0.05
TT (ng/mL), median (range)	0.12 (0.05–2.71)	0.2 (0.05–2.71)*	0.12 (0.05–1.33)	0.08 (0.05–0.56)*	< 0.05
Hb (g/dL), median (range)	10.9 (7.2–14.8)*	10.8 (8.5–14.4)	10.6 (7.2–13.0)*	11.9 (8.8–14.8)*	< 0.05
AP (U/L), median (range)	171 (34 − 3,057)	171 (34 − 3,057)	179 (73–986)	125 (40 − 1,513)	
LDH (U/L), median (range)	348 (164-3,934)	308 (164-2,748)	472 (189-1,781)	337 (172-3,934)	
> ULN, no. (%)	92 (74.8)	47 (72.3)	24 (77.4)	21 (77.8)	
> 2x ULN, no. (%)	40 (32.5)	17 (26.2)*	15 (48.4)*	8 (29.6)	< 0.05
CRP (mg/L), median (range)	6.8 (1-169.6)*	3.7 (1–95.0)*	15.0 (1-169.6)*	8.25 (1–60.0)	< 0.05
> ULN, no. (%)	63/111 (56.8)*	25/58 (43.1)*	19/29 (65.5)*	19/24 (79.2)*	< 0.05
NSE (µg/L), median (range)	20 (8-411)	17 (8-189)*	27 (12–300)*	26 (11–411)	< 0.05
> ULN, no. (%)	78/117 (66.7)	31/59 (52.5)*	26 (83.9)*	21 (77.8)*	< 0.05
CgA (µg/L) > ULN, no. (%)	71/116 (61.2)	37/58 (63.8)	19 (61.3)	15 (55.6)	
NLR, median (range)	5.7 (1.58–40.6)	6.0 (1.79–31.67)	4.9 (2.03–18.67)	6.6 (1.58–40.6)	

*mDRPC* Metastatic docetaxel-resistant prostate cancer, *ART* androgen-receptor targeted therapy, *FT* free testosterone, *DL* detection limit, *ECOG* Eastern Cooperative Oncology Group, *PSA* prostate-specific antigen, *TURP* transurethral resection of the prostate, *DC* docetaxel plus carboplatin, *PSMA* prostate-specific membrane antigen, *ADT* androgen deprivation therapy, *TT* total testosterone, *Hb* hemoglobin, *AP* alkaline phosphatase, *LDH* lactate dehydrogenase, *ULN* upper limit of normal, *CRP* c-reactive protein, *NSE* neuron-specific enolase, *CgA* chromogranin A, *NLR* neutrophil-to-lymphocyte ratio. All groups showing statistically significant differences (by using t-tests und chi-square tests) were marked with an asterisk.

Patients in group 3, in contrast, had a significantly longer prior ADT duration and a significantly lower rate of primary resistance to docetaxel (25.9%).

The need for non-narcotic pain relief was significantly highest in group 2 (74.2%), compared to patients of group 1 (43.1%) and 3 (48.1%), although the overall presence of pain was similar across the groups.

Regarding laboratory values at baseline, patients in group 2 had significantly lower Hb levels and exhibited significantly the highest serum levels of NSE, CRP, and LDH. By contrast, group 3 patients had significantly higher serum Hb levels.

### Effects on testosterone

**Fig. 1:**
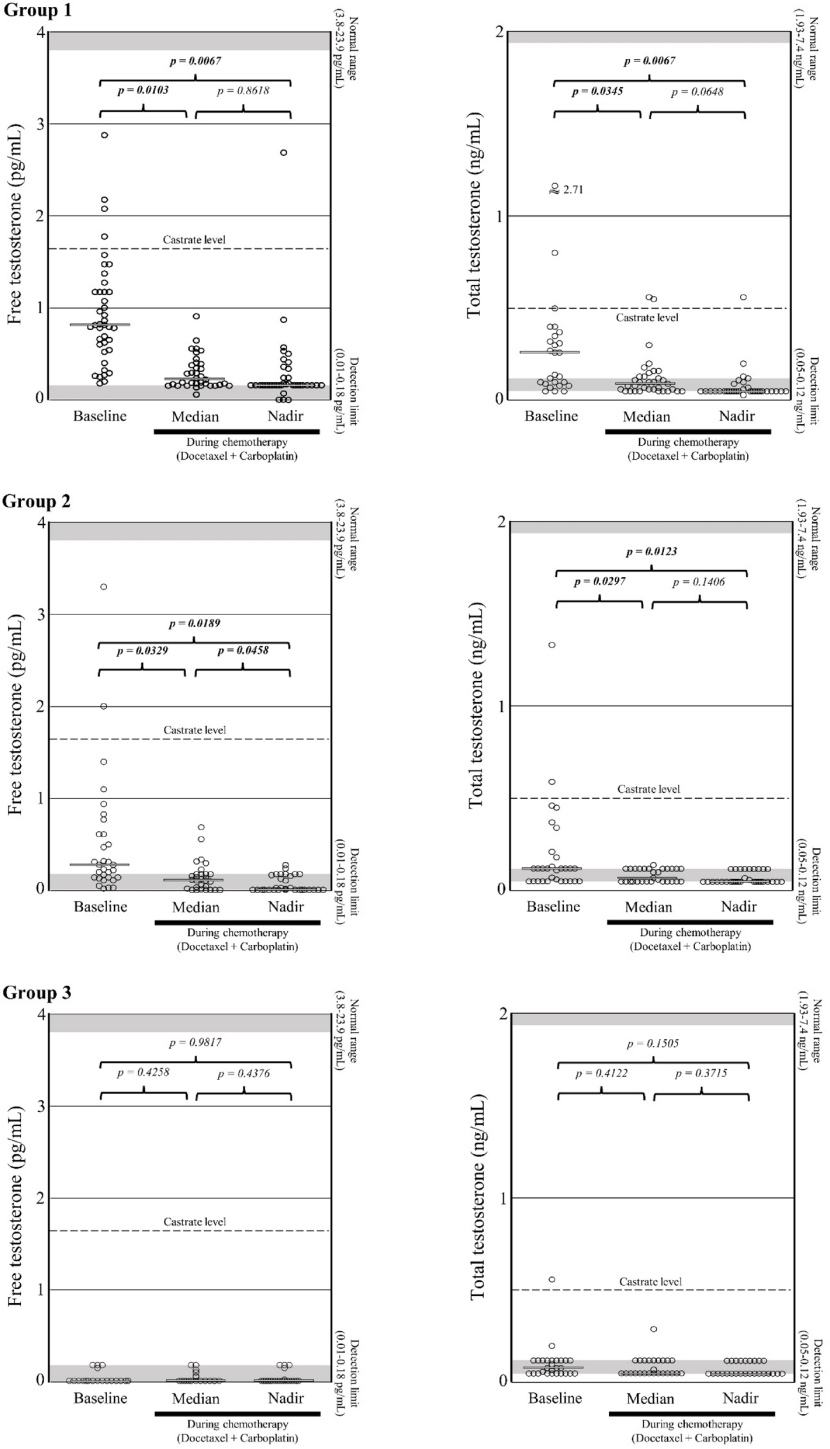
Baseline, median and nadir values of free testosterone (FT) and total testosterone (TT) levels for patients of the groups 1 (no previous androgen-receptor targeted therapy (ART)), 2 (previous ART and FT > DL at baseline) and 3 (previous ART and FT < DL at baseline). Castrate level for FT is defined as 1.7 pg/mL^32^ and for TT as 0.5 ng/mL^24^. The gray bars represent the median values of each category.

**Fig. 2:**
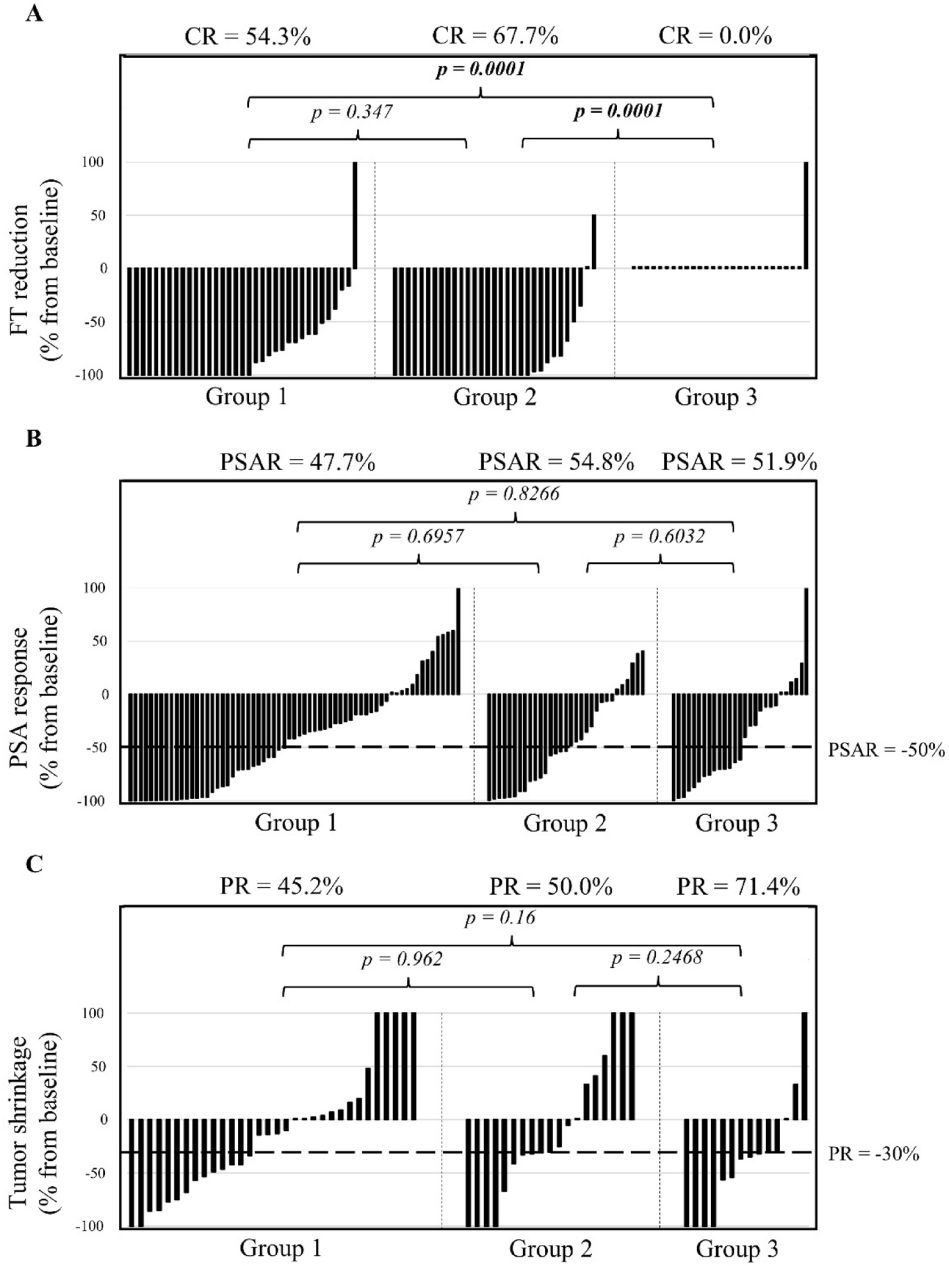
Response to docetaxel plus carboplatin. (**A**) Free testosterone (FT) reduction. *CR* Complete reduction. (**B**) Prostate-specific antigen response (PSAR). PSAR is defined as a ≥ 50% decline in PSA from baseline according to Prostate Cancer Working Group (PCWG) 2/3 criteria^24,26^. (**C**) Shrinkage of soft tissue tumor metastases. Partial response (PR) is defined in the Response Evaluation Criteria in Solid Tumors (RECIST) guideline version 1.1 as a ≥ 30% decrease in the sum of diameters of target lesions, taking as reference the baseline sum diameters^25^.

Salvage DC chemotherapy in group 1 led to a decrease from a median of 0.85 pg/mL at baseline to nadir values below the DL (< 0.18 pg/mL) (*p* = 0.0067; Tables 1and 2; Fig.1). 54.3% of the patients in this group had a complete FT reduction (CR = -100%) (Table 2; Fig. 2A).

**Table 2:** Response to treatment, disease progression and adverse events.

	All (mDRPC) *n* = 123	Group 1 (mDRPC w/o ART) *n* = 65	Group 2 (mDRPC + ART: FT > DL) *n* = 31	Group 3 (mDRPC + ART: FT < DL) *n* = 27	*p* value*
No. of DC cycles, median (range)	8 (2-60)	8 (2-60)	6 (2-37)	8 (2-36)	
Post-DC treatment, no. (%)					
Cabazitaxel	13 (10.6)	10 (15.4)	2 (6.5)	1 (3.7)	
Abiraterone	20 (16.3)*	19 (29.2)*	0 (0.0)*	1 (3.7)*	< 0.05
**Response to treatment**					
FT (pg/mL)	n = 100	n = 42	n = 31	n = 27	
Median, median	0.16*	0.25*	0.11*	0.01*	< 0.05
(range)	(0.01–0.93)	(0.06–0.93)	(0.01–0.69)	(0.01–0.26)	
Nadir, median	0.13*	0.18*	0.01*	0.01*	< 0.05
(range)	(0.01–2.7)	(0.01–2.7)	(0.01–0.28)	(0.01–0.18)	
Time to nadir (days) , median	49.5				
(95% CI)	(6.3–92.7)				
Reduction (%), median	-77.5*	-100*	-100*	0.0*	< 0.05
(95% CI)	(-88.5 to -66.5)	(-116.0 to -84.0)	(-111.7 to -88.3)	(-7.1 to + 7.1)	
Reduction = -100%, no. (%)	38/93 (40.9)*	19/35 (54.3)*	21 (67.7)*	0 (0.0)*	< 0.05
TT (ng/mL)	n = 83	n = 26	n = 30	n = 27	
Median, median	0.07	0.09	0.07	0.05	
(range)	(0.05–0.56)	(0.05–0.56)	(0.05–0.14)	(0.05–0.29)	
Nadir, median	0.05	0.05	0.05	0.05	
(range)	(0.03–0.56)	(0.03–0.56)	(0.05–0.12)	(0.05–0.12)	
Reduction (%), median	0*	-100*	0*	0*	< 0.05
(95% CI)	(-10.9 to + 10.9)	(-116.9 to -83.1)	(-16.8 to + 16.8)	(-17.0 to + 17.0)	
Reduction = -100%, no. (%)	30/76 (39.5)	11/20 (55.0)	10/30 (33.3)	7/26 (26.9)	
PSA					
Response, no. (%)	62 (50.4)	31 (47.7)	17 (54.8)	14 (51.9)	
Time to response (days), median	56				
(95% CI)	(42.8–69.2)				
Reduction (%), median	-48.6	-41.7	-53.4	-61.4	
(95% CI)	(-57.7 to -39.5)	(-54.4 to -29.0)	(-68.9 to -37.9)	(-82.8 to -40.0)	
Pain response, no. (%)	55/122 (45.1)*	21 (32.3)*	20/30 (66.7)*	14 (51.9)	< 0.05
Radiographic response					
Bone metastasis, no. (%)	n = 119	n = 61	n = 31	n = 27	
Not available (thereof early death)	13 (7)	6 (5)	3 (1)	4 (1)	
Improvement	13/106 (12.3)	6/55 (10.9)	4/28 (14.3)	3/23 (13.0)	
Stable	75/106 (70.8)	39/55 (70.9)	18/28 (64.3)	18/23 (78.3)	
Progression	15/106 (14.2)	8/55 (14.5)	5/28 (17.9)	2/23 (8.7)	
Soft tissue metastasis	n = 78	n = 37	n = 23	n = 18	
Not available (thereof early death)	13 (7)	6 (5)	3 (1)	4 (1)	
RECIST (%), median	-30.0	-13.5	-30.0	-35.7	
(95% CI)	(-45.1 to -14.9)	(-34.3 to + 7.3)	(-60.0 to 0.0)	(-64.3 to -7.1)	
PR, no. (%)	34/65 (52.3)	14/31 (45.2)	10/20 (50.0)	10/14 (71.4)	
SD, no. (%)	17/65 (26.2)	12/31 (38.7)	3/20 (15.0)	2/14 (14.3)	
PD, no. (%)	14/65 (21.5)	5/31 (16.1)	6/20 (30.0)	3/14 (21.4)	
**Disease progression**					
PFS (months), median	6.9	6.8	6.8	7.6	
(95% CI)	(6.2–7.6)	(5.2–8.4)	(5.5–8.1)	(4.4–10.7)	
OS (months), median	14.4	19.2*	11.9*	13.5*	< 0.05
(95% CI)	(12.1–16.8)	(13.1–25.2)	(8.7–15.2)	(10.0–16.9)	
OS w/o post-DC treatment (months), median	11.9	11.5	10.9	12.9	
(95% CI)	(9.5–14.4)	(6.7–16.4)	(6.2–15.7)	(7.7–18.2)	
**Adverse events**					
Hematological, no. (%)					
Anemia					
Grade 1 + 2	86 (69.9)	50 (76.9)*	17 (54.8)*	19 (70.4)	< 0.05
Grade 3 + 4	35 (28.5)	15 (23.1)	13 (41.9)	7 (25.9)	
Blood transfusion	79 (64.2)	42 (64.6)	22 (71.0)	15 (55.6)	
Leukopenia					
Grade 1 + 2	58 (47.2)	30 (46.2)	11 (35.5)*	17 (63.0)*	< 0.05
Grade 3 + 4	48 (39.0)	25 (38.5)	15 (48.4)	8 (29.6)	
Neutropenia					
Grade 1 + 2	30 (24.4)	11 (16.9)*	11 (35.5)*	8 (29.6)	< 0.05
Grade 3 + 4	41 (33.3)	24 (36.9)	11 (35.5)	6 (22.2)	
G-CSF	22 (17.9)	7 (10.8)	8 (25.8)	7 (25.9)	
Thrombopenia					
Grade 1 + 2	64 (52.0)	32 (49.2)	15 (48.4)	17 (63.0)	
Grade 3 + 4	26 (21.1)	13 (20.0)	9 (29.0)	4 (14.8)	
Non-hematological, no. (%)					
Fatigue	98 (79.7)	47 (72.3)	27 (87.1)	24 (88.9)	
Dyspnea	70 (56.9)	37 (56.9)	15 (48.4)	18 (66.7)	
Nausea	52 (42.3)	25 (38.5)	13 (41.9)	14 (51.9)	
Polyneuropathy	43 (35.0)*	34 (52.3)*	6 (19.4)*	3 (11.1)*	< 0.05
Nail changes	27 (22.0)*	21 (32.3)*	5 (16.1)	1 (3.7)*	< 0.05
Constipation	19 (15.4)	12 (18.5)	2 (6.5)	5 (18.5)	
Infection	17 (13.8)	9 (13.8)	5 (16.1)	3 (11.1)	

*mDRPC* Metastatic docetaxel-resistant prostate cancer,* ART* androgen-receptor targeted therapy,* FT* free testosterone,* DL* detection limit,* DC* docetaxel plus carboplatin,* TT* total testosterone,* PSA* prostate-specific antigen,* RECIST * Response Evaluation Criteria In Solid Tumors,* PR* partial response,* SD* stable disease,* PD* progressive disease,* PFS *progression-free survival,* CI* confidence interval,* OS* overall survival,* G-CSF* granulocyte-colony stimulating factor. All groups showing statistically significant differences (by using t-tests und chi-square tests) were marked with an asterisk.

In group 2, DC chemotherapy resulted in FT decreasing from a median of 0.28 pg/mL at baseline to nadir values below the DL (< 0.18 pg/mL) (*p* = 0.0189; Tables 1  and 2; Fig. 1). 67.7% of the patients had a CR (Table 2; Fig. 2A).

In group 3, the median FT baseline, median and nadir values were below the DL (< 0.18 pg/mL) (Tables 1 and 2; Fig. 1). As a consequence, no further FT reduction could be observed (Table 2 Fig. 2A). Additionally, all of the FT values in group 3 were significantly lower than those in group 1 and 2 (*p* < 0.05; Tables 1 and 2). In 26 out of 27 patients (96.3%), the nadir value was below the DL. This is a highly significant difference compared to group 1 (65.8%; *p* = 0.0032) and group 2 (64.5%; *p* = 0.0029) (data not shown).

The median time to FT nadir was 49.5 days (95% CI 6.3–92.7) across the entire study cohort (Table 2).

Considering TT, salvage DC chemotherapy resulted in nadir values below the DL (< 0.12 ng/mL) in all three groups (Table 2 Fig. 1). The median TT baseline values were 0.2 ng/mL (group 1), 0.12 ng/mL (group 2) and 0.08 ng/mL (group 3). In this regard, the baseline value of group 3 was significantly lower (*p* < 0.05) compared to that of group 1 (Table 1; Fig. 1). A -100% reduction in TT levels was observed in 55.0% (group 1), 33.3% (group 2) and 26.9% (group 3) of the patients (Table 2).

### Patient outcome

In the overall study population, patients received a median of 8 consecutive cycles of DC chemotherapy (range 2–60). Potential post-DC treatment consisted of cabazitaxel and/or abiraterone. Patients of group 1 (without prior ART) received post-DC abiraterone substantially more frequently than patients of the groups 2 and 3 (with prior ART) (Table 2).

In the general study population, a PSAR (≥ 50% decline in PSA from baseline) was observed in 62 out of 123 patients (50.4%). The median PSA reduction was − 48.6% (95% CI -57.7 – -39.5). The median time to PSAR was 56 days (95% CI 42.8–69.2) (Table 2).

In terms of PSAR, there were no considerable differences between the groups 1, 2 and 3 (Fig. 2B).

Overall, 34 out of 65 available patients (52.3%) with measurable disease experienced a partial response (PR) (Table 2). Seventeen out of 65 patients (26.2%) had a stable disease (SD), while 14 out of 65 patients (21.5%) had a progressive disease (PD). The median shrinkage of soft tissue metastases was − 30% (95% CI -45.1 – -14.9) (Table 2). Out of the 106 available patients with bone metastasis at baseline, 13 (12.3%) showed an improvement in the bone scans, 75 (70.8%) exhibited no change and 15 (14.2%) experienced disease progression (Table 2). Regarding radiographic response, no substantial differences were observed between the groups 1, 2 and 3 (Table 2; Fig. 2C).

**Fig. 3:**
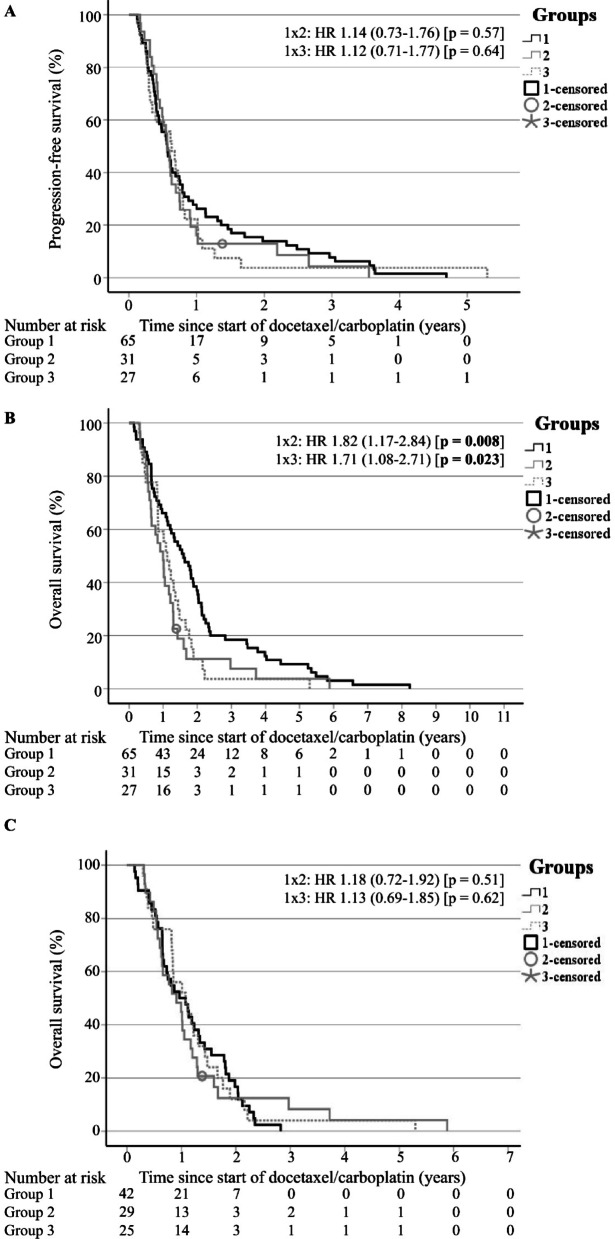
Kaplan-Meier curves for patients of the groups 1 (black line), 2 (gray line) and 3 (gray dotted line). *HR* Hazard ratio. (**A**) Progression-free survival. (**B**) Overall survival. (**C**) Overall survival without post-docetaxel plus carboplatin treatment (i.e., cabazitaxel and/or abiraterone).

The median follow-up time for all patients was 14.4 months (95% CI 11.3–17.5) (data not shown). The median PFS was 6.9 months (95% CI 6.2–7.6) and did not differ considerably between the three groups (Table 2; Fig. 3A). Median OS for all patients was 14.4 months (95% CI 12.1–16.8) (Table 2). Patients of group 1 (without prior ART) had a significantly longer OS (median of 19.2 months, 95% CI 13.1–25.2) compared to those with prior ART (group 2: median OS of 11.9 months, 95% CI 8.7–15.2; group 3: median OS of 13.5 months, 95% CI 10.0–16.9; *p* = 0.008 and 0.023, respectively) (Table 2 Fig. 3B). The significant OS advantage of group 1 was eliminated by excluding patients who underwent post-DC treatment (cabazitaxel and/or abiraterone) (Table 2; Fig. 3C).

### Multivariate analyses

Univariate analyses of baseline and treatment-dependent parameters associated with PFS and OS are shown in Supplementary Table S1.

In multivariate analyses with stepwise regression, CRP > upper limit of normal (ULN), PSAR and FT nadir < 0.2 pg/mL were independent predictors for PFS in the general study population (Table 3). In group 1 (no prior ART), PSAR and FT nadir < 0.2 pg/mL were independent predictors for a better PFS. Group 2 (prior ART and FT > DL) revealed primary resistance towards docetaxel, CRP > ULN, PSAR and FT median < 0.18 pg/mL as independent prognostic factors for PFS. In contrast, independent prognostic PFS indicators in group 3 (prior ART and FT < DL) were the baseline parameters LDH > 2x ULN and presence of pain.

**Table 3:** Baseline and treatment-dependent parameters influencing progression-free survival, overall survival and PSA response: multivariate analyses.

	All (mDRPC) *n* = 123	Group 1 (mDRPC w/o ART) *n* = 65	Group 2 (mDRPC + ART: FT > DL) *n* = 31	Group 3 (mDRPC + ART: FT < DL) *n* = 27
	Hazard ratio (95% confidence interval) *p* value (adjusted *p* value using the Benjamini-Hochberg formula)			
**Progression-free survival**				
*Baseline parameters*				
Primary resistant towards docetaxel			10.35 (3.25–32.96) *< 0.001 (0.011)*	
CRP > ULN	2.11 (1.35–3.3) *< 0.001 (0.011)*		3.79 (1.42–10.15) *0.008# (0.0266)*	
LDH > 2x ULN				13.52 (3.72–49.19) *< 0.001 (0.011)*
Presence of pain				0.13 (0.04–0.47) *< 0.001 (0.011)*
*Treatment-dependent parameters*				
PSA response	0.27 (0.17–0.44) < 0.001* (0.011)	0.18 (0.08–0.43) *< 0.001 (0.011)*	0.16 (0.06–0.46) *< 0.001 (0.011)*	
FT nadir < 0.2 pg/mL	0.37 (0.18–0.75) *0.006 (0.033)*	0.3 (0.12–0.79) *0.014# (0.0308)*		
FT median < 0.18 pg/mL			0.19 (0.05–0.7) *0.012# (0.033)*	
**Overall survival**				
*Baseline parameters*				
CRP > ULN	2.27 (1.22–4.24) *0.01# (0.0217)*			
LDH > 2x ULN	2.45 (1.34–4.47) *0.003 (0.013)*	4.6 (1.96–10.81) *< 0.001 (0.013)*		4.82 (1.89–12.28) *< 0.001 (0.013)*
NSE > 3x ULN	1.96 (1.01–3.8) *0.046# (0.0854)*			
Progression of bone metastasis only	0.51 (0.27-1.0) *0.049# (0.0793)*			
Pulmonary metastasis				4.12 (1.42–11.9) *0.009# (0.0234)*
Non-narcotics required			4.03 (1.48–11.01) *0.007# (0.0228)*	
*Treatment-dependent parameters*				
PSA response	0.4 (0.24–0.66) < 0.001** (0.013)		0.19 (0.07–0.47) *< 0.001 (0.013)*	
FT nadir < 0.2 pg/mL		0.19 (0.08–0.47) *< 0.001 (0.013)*		
FT median < detection limit		0.17 (0.05–0.53) *0.002 (0.013)*		
TT median < detection limit	2.63 (1.57–4.41) *< 0.001 (0.013)*			
**PSA response**				
*Baseline parameters*				
LDH > ULN	2.68 (1.06–6.78) *0.037# (0.037)*			
Narcotics required				7.33 (1.16–46.24) *0.034# (0.0425)*
*Treatment-dependent parameters*				
Pain response	4.73 (2.15–10.41) *< 0.001 (0.005)*			
Flare phenomenon			0.13 (0.03–0.72) *0.019# (0.0317)*	
FT median < 0.2 pg/mL		10 (1.81–55.28) *0.008 (0.02)*		

*mDRPC* Metastatic docetaxel-resistant prostate cancer, *ART* androgen-receptor targeted therapy, *FT* free testosterone, *DL* detection limit, *CRP* c-reactive protein, *ULN* upper limit of normal, *LDH* lactate dehydrogenase, *PSA* prostate-specific antigen, *NSE* neuron-specific enolase, *TT* total testosterone. Non-significant p values after Bonferroni correction for multiple hypothesis testing are marked with #, with adjusted significance thresholds calculated as 0.0071 for progression-free survival, 0.005 for overall survival, and 0.01 for PSA response. Applying ln(T), time-dependent covariate analysis revealed significant time-dependence for PSA response: *p = 0.024 for progression-free survival, **p = 0.042 for overall survival.

Regarding OS in the general study population, multivariate analyses with stepwise regression identified CRP > ULN, LDH > 2x ULN, NSE > 3x ULN, progression of bone metastasis only, PSAR and TT median < DL as independent predictors (Table3) For the subgroups, the following independent prognostic factors remained for OS: LDH > 2x ULN, FT nadir < 0.2 pg/mL and FT median < DL (group 1); requirement for non-narcotics and PSAR (group 2); LDH > 2x ULN and pulmonary metastasis (group 3).

By applying extended time-dependent covariate analyses (T; ln(T); T ≥ 365 days), log(-log (survival probability)) and conditional landmark analyses, it was shown that all treatment-dependent prognostic parameters (e.g., PSAR, FT and TT levels) had no association with time, except for ln(T) for PSAR in the general study population. This particular covariate revealed significant time-dependence (*p* = 0.024 for PFS and 0.042 for OS, respectively) (data not shown).

Binary logistic regression analyses demonstrated an association of LDH > ULN and pain response with a favorable PSAR in the overall study population. In group 1, FT median < 0.2 pg/mL was linked to a better PSAR. In group 2, flare phenomenon was associated with a worse PSAR. Lastly, the requirement for narcotics in group 3 was linked to an improved PSAR.

### Adverse events

Dose reductions due to treatment-related toxicities were common (71/123, 57.7%), mainly attributed to hematotoxicity (data not shown). Hematological grade 3 + 4 toxicities included anemia (28.5%), leukopenia (39.0%), neutropenia (33.3%) and thrombopenia (21.1%). Seventy-nine out of 123 patients (64.2%) received one or more blood transfusions during DC treatment (Table 2).

Treatment-related non-hematological toxicities included fatigue, dyspnea, nausea, polyneuropathy, nail changes, constipation and infection (Table 2).

Overall, hematological and non-hematological adverse events were well balanced between the groups (Table 2). However, patients of group 1 experienced grade 1 or 2 anemia significantly more frequently than patients of group 2 (76.9% vs. 54.8%, *p* < 0.05). Between these groups a significant difference was also observed for grade 1 or 2 neutropenia: 16.9% (group 1) vs. 35.5% (group 2) (*p* < 0.05). Regarding polyneuropathy and nail changes, patients of group 1 were significantly more affected than those of the other groups (*p* < 0.05). Finally, patients of group 3 experienced grade 1 or 2 leukopenia significantly more frequently than patients of group 2 (63.0% vs. 35.5%, *p* < 0.05). Grade 3 or 4 anemia, leukopenia and neutropenia did not differ significantly (Table 2).

## Discussion

In this study, we demonstrate that serum FT and TT levels are reduced during DC chemotherapy in mCRPC patients. These results are consistent with the observations previously published by our group showing that serum FT and TT levels were reduced during first-line docetaxel chemotherapy in both mCNPC and mCRPC patients^5^.

Furthermore, our study highlights that a FT nadir level < 0.2 pg/mL serves as an independent predictor for PFS and a TT median level < DL is an independent predictor for OS in our overall study population. Our results suggest that whenever FT baseline values are > DL, the treatment-induced suppression of FT plays a significant prognostic role for the treatment outcome. In particular, in ART-naive patients (i.e., group 1), low FT treatment values are independent prognostic predictors for PFS, OS and PSAR (Table 3). In patients with prior ART and FT baseline values > DL, a low FT treatment value (i.e., FT median < 0.18 pg/mL) is an independent prognostic parameter for PFS. In contrast, low testosterone treatment values hold no prognostic value in patients with prior ART and FT baseline values < DL (Table 3).

Recently, we had also uncovered a major biological role of testosterone in docetaxel treatment outcomes of mCNPC and mCRPC patients^5^. However, we found that only FT nadir < DL and complete FT suppression during docetaxel treatment were significant predictors for PFS, while complete FT suppression alone was a predictor for OS^5^.

Other groups had reported that mCRPC patients who experienced a high decline in TT (greater than the median) during docetaxel chemotherapy had a significantly higher median OS of 26.3 months (95% CI 23.4–28.1) compared to patients with no or minimal decline in TT, who had a median OS of 20.9 months (95% CI 19.3–23.4; *p* = 0.003)^10^.

Additionally, our data indicate that the efficacy of DC chemotherapy (e.g., PSAR rate, radiographic response rate and PFS) is similar in mDRPC patients with or without prior ART treatment. Specifically, the PSAR rate was 53.4% (31 out of 58 patients) and the median PFS was 7.2 months in patients with prior ART, compared to a PSAR rate of 47.7% (31 out of 65 patients) and a median PFS of 6.8 months in patients without prior ART.

The efficacy of cabazitaxel chemotherapy in mCRPC patients was also reported to be similar regardless of prior ART treatment^11,33–37^. The PSAR rates for cabazitaxel after prior ART ranged between 32 and 39%, with a median PFS between 4.4 and 6.5 months and a median OS between 10.9 and 17.0 months^37^. Similar data were reported for cabazitaxel in ART-naive patients in the TROPIC trial, with a PSAR rate of 39%, a median PFS of 2.8 months and a median OS of 15.1 months^38^. Furthermore, the PROSELICA trial conveyed a PSAR rate of 42.9%, a median PFS of 3.5 months and a median OS of 14.5 months for 25 mg/m² cabazitaxel q3w in mostly ART-naive patients^39^.

These findings contrast with reports indicating a decrease in the efficacy of docetaxel chemotherapy to 26–50% in ART-pretreated compared to ART-naive mCRPC patients^5,11,12,40–44^. In ART-naive patients, the PSAR rate was 63% in the VENICE trial and 68.4% in the FIRSTANA trial^45,46^. Similarly, recent reports indicated a PSAR rate of 63% and a median PFS of 7.6 months in mCRPC patients who received docetaxel alone. In contrast, the PSAR rate was 38% and the median PFS was 4.4 months in mCRPC patients who received docetaxel after previous treatment with abiraterone (*p* = 0.02 and *p* = 0.003, respectively)^41^.

Docetaxel clearance in prostate cancer patients has been reported as castration-dependent, with castrated men experiencing a 100% increase in clearance and a two-fold reduction in area under the curve, despite unchanged hepatic CYP3A4 activity. Conversely, castration-naive patients had higher drug exposure and more severe hematotoxicity. A recent study also found that lower baseline testosterone levels led to lower intracellular docetaxel levels and reduced treatment response rates^47^.

Beyond testosterone levels, the presence of molecular alterations may affect the effectiveness of chemotherapy in mCRPC. Combined tumor suppressor gene defects involving TP53, PTEN, and RB1 characterize clinically defined aggressive variant prostate cancers (AVPC)^48^. The presence of at least one of the seven AVPC clinicopathological criteria was associated with a high proportion of patients responding to carboplatin and docetaxel, irrespective of morphology^49^.

Furthermore, sensitivity to platinum-based chemotherapy in men with mCRPC has been shown to be associated with germline mutations in BRCA2^50,51^. In a large retrospective study of patients with mCRPC treated with platinum-based chemotherapy, response rates were higher in the subgroup of 44 patients with BRCA2 gene alterations, with a PSAR of at least 50% in 23 patients (63.9%) and soft tissue responses in 17 patients (38.6%) with evaluable disease^52^. However, contrasting findings were reported in another study, where the addition of carboplatin to cabazitaxel was not beneficial for the 36 men (40%) with BRCA2 somatic alterations detected in circulating tumor DNA^16^. In line with the findings of Corn et al., Van der Zande et al. observed that patients harboring mutations in genes of interest (BRCA1, BRCA2, ATM, TP53, RB1, or PTEN) exhibited a similar PSAR to combination treatment as patients without these mutations^53^. Van der Zande et al. combined cabazitaxel and carboplatin for the treatment of mCRPC patients with innate or acquired resistance to cabazitaxel monotherapy, with 12 patients (26.6%) experiencing a PSA decline of ≥ 50% from baseline^53^. In our study with DC, 62 out of 123 mDRPC patients (50.4%) demonstrated a PSAR ≥ 50% (Table 2).

Our study is limited by its retrospective non-randomized design and potential selection bias. Additionally, it may have lacked the power to separately analyze outcomes for our groups. Most reports on sequence of treatment in prostate cancer are retrospective and are susceptible to lead-time bias. Patients with prior ART treatment had more advanced disease at baseline than ART-naive patients. To mitigate potential lead-time bias, we applied extended time-dependent Cox modelling, log(-log(survival probability)) plots for each covariate level and conditional landmark analyses. Using PCWG criteria, disease progression was primarily attributed to PSA progression (e.g., PSA progression or radiographic progression, whichever occurred first). Scanning intervals for evaluating progression were not uniformly adhered to, and generally, confirmatory scans were not conducted.

## Conclusion

This study demonstrates that adding carboplatin to docetaxel chemotherapy may overcome docetaxel resistance in approximately 50% of mDRPC patients, regardless of prior ART, contrasting with reported reductions in efficacy for docetaxel in ART-pretreated patients. Furthermore, serum FT and TT levels were reduced during DC chemotherapy and are independent predictors of PSAR, PFS, and OS. However, when FT values were < DL in ART-pretreated mDRPC patients, testosterone was not linked to clinical outcomes. Our findings indicate that the addition of carboplatin to docetaxel chemotherapy may improve the therapeutic effects of docetaxel in a castration-dependent setting.

## Electronic supplementary material

Below is the link to the electronic supplementary material.

Supplementary Material 1

## Data Availability

The datasets generated during and/or analyzed during the current study are available from the corresponding author on reasonable request.
