# Supplementary material for: Impact of adding carboplatin to docetaxel chemotherapy on testosterone levels and treatment outcomes in metastatic docetaxel-resistant prostate cancer
Source: Sci Rep. 2025 Jun 20;15:20130. doi: 10.1038/s41598-025-04667-0 (PMC12181351; doi:10.1038/s41598-025-04667-0)
Supplement: Supplementary file 1 — Supplementary Material 1 [file 41598_2025_4667_MOESM1_ESM.pdf]

Impact of adding carboplatin to docetaxel chemotherapy on testosterone levels and treatment outcomes in metastatic docetaxel-resistant prostate cancer.

Hejar Atalan[1], Michael A. Morgan[2], Philipp Ivanyi[1], Paula Kappler[1], Florian H. Heidel[1], and Christoph W. M. Reuter[1,*]

[1]Department of Hematology, Hemostasis, Oncology and Stem Cell Transplantation, Hannover Medical School, Carl-Neuberg-Str. 1, 30625 Hannover, Germany.
[2]Institute of Experimental Hematology, Hannover Medical School, Carl-Neuberg-Str. 1, 30625 Hannover, Germany.

Supplementary Table S1 - Baseline and treatment-dependent parameters influencing progression-free survival, overall survival and PSA response: univariate analyses

	All (mDRPC)<br><br>n = 123	Group 1 (mDRPC w/o ART)<br>n = 65	Group 2 (mDRPC+ART: FT >DL)<br>n = 31	Group 3 (mDRPC+ART: FT <DL)<br>n = 27
	Hazard ratio (95% confidence interval)<br>*p value*			
**Progression-free survival**				
Baseline parameters				
Number of previous chemotherapies >1	1.65 (1.05-2.59)<br>*0.031*	2.1 (1.08-4.09)<br>*0.029*		
Prostatectomy			2.42 (1.11-5.27)<br>*0.027*	
Primary resistant towards docetaxel			2.99 (1.3-6.88)<br>*0.01*	
CRP >ULN	1.84 (1.24-2.73)<br>*0.002*		3.11 (1.26-7.71)<br>*0.014*	
LDH >2x ULN	1.98 (1.34-2.92)<br>*<0.001*			4.76 (1.76-12.85)<br>*0.002*
NSE >ULN	1.55 (1.04-2.3)<br>*0.03*			
Hb <10 g/dL	2.26 (1.47-3.47)<br>*<0.001*	2.52 (1.33-4.77)<br>*0.005*	2.3 (1.04-5.06)<br>*0.039*	
AP >ULN			2.36 (1.04-5.37)<br>*0.041*	
Progression of bone metastasis	0.61 (0.42-0.9)<br>*0.012*		0.4 (0.17-0.91)<br>*0.029*	
Progression of bone metastasis only	0.58 (0.38-0.88)<br>*0.011*	0.56 (0.32-0.97)<br>*0.038*	0.38 (0.15-0.96)<br>*0.04*	
Progression of soft tissue metastasis only			2.31 (1-5.36)<br>*0.05*	
Soft tissue metastasis (RECIST)			2.71 (1.08-6.84)<br>*0.035*	
Number of involved organs = 1			0.26 (0.09-0.78)<br>*0.017*	
Number of involved organs ≥3	1.64 (1.06-2.51)<br>*0.025*	1.85 (1.01-3.38)<br>*0.046*		
Number of involved soft tissue organs	1.27 (1.04-1.57)<br>*0.022*		1.67 (1.08-2.58)<br>*0.022*	
Presence of pain				0.37 (0.15-0.93)<br>*0.035*
Non-narcotics required			2.59 (1.03-6.5)<br>*0.043*	
Treatment-dependent parameters				
PSA response	0.27 (0.18-0.4)<br>*<0.001*	0.18 (0.1-0.33)<br>*<0.001*	0.32 (0.15-0.71)<br>*0.005*	
Pain response	0.58 (0.41-0.84)<br>*0.004*	0.51 (0.3-0.87)<br>*0.013*		
Flare phenomenon			2.25 (1.01-4.98)<br>*0.046*	
FT reduction >-50%		0.24 (0.09-0.69)<br>*0.008*		
FT nadir <0.2 pg/mL	0.34 (0.18-0.64)<br>*<0.001*	0.17 (0.07-0.42)<br>*<0.001*		
FT nadir <0.18 pg/mL	0.57 (0.33-1.0)<br>0.048	0.33 (0.15-0.69)<br>*0.004*		
FT nadir <detection limit	0.62 (0.39-0.98)<br>*0.041*	0.33 (0.15-0.69)<br>*0.004*		
FT median <0.2 pg/mL		0.44 (0.22-0.88)<br>*0.021*	0.28 (0.11-0.76)<br>*0.012*	
FT median <0.18 pg/mL		0.38 (0.17-0.87)<br>*0.021*	0.28 (0.11-0.76)<br>*0.012*	
FT median <detection limit		0.29 (0.12-0.73)<br>*0.008*		
**Overall survival**				
Baseline parameters				
ECOG >1	1.7 (1.13-2.57)<br>*0.012*	2.02 (1.13-3.6)<br>*0.017*		
Number of previous chemotherapies >1	1.87 (1.19-2.95)<br>*0.007*	2.31 (1.18-4.53)<br>*0.015*		
Prostatectomy		0.6 (0.36-0.99)<br>*0.044*		

Radiotherapy of soft tissue	1.61 (1.04-2.52)<br>*0.035*			
PSA >67 µg/L (median) at castration-resistant status		2.02 (1.21-3.36)<br>*0.007*		
CRP >ULN	2.49 (1.64-3.77)<br>*<0.001*	2.06 (1.18-3.59)<br>*0.011*	2.54 (1.05-6.15)<br>*0.039*	
LDH >2x ULN	2.78 (1.87-4.13)<br>*<0.001*	2.46 (1.39-4.36)<br>*0.002*		4.0 (1.63-9.8)<br>*0.002*
NSE >ULN	1.73 (1.16-2.58)<br>*0.007*			
NSE >2x ULN	1.78 (1.17-2.7)<br>*0.007*		2.29 (1.05-5.0)<br>*0.037*	
NSE >3x ULN	1.97 (1.18-3.29)<br>*0.009*		2.61 (1.12-6.08)<br>*0.027*	
Hb <10 g/dL	2.92 (1.88-4.53)<br>*<0.001*	2.94 (1.53-5.66)<br>*<0.001*		5.47 (1.71-17.49)<br>*0.004*
Progression of bone metastasis	0.66 (0.45-0.97)<br>*0.032*			
Progression of soft tissue metastasis	1.76 (1.21-2.55)<br>*0.003*			
Progression of bone and soft tissue metastasis		1.97 (1.16-3.34)<br>*0.012*		
Progression of bone metastasis only	0.43 (0.28-0.68)<br>*<0.001*	0.4 (0.23-0.72)<br>*0.002*		
Progression of soft tissue metastasis only	1.6 (1.0-2.54)<br>*0.049*		3.27 (1.38-7.76)<br>*0.007*	4.35 (1.47-12.93)<br>*0.008*
Soft tissue metastasis (RECIST)	1.87 (1.27-2.75)<br>*0.002*	1.82 (1.09-3.05)<br>*0.023*		
Number of involved organs = 1	0.49 (0.33-0.73)<br>*<0.001*	0.54 (0.32-0.9)<br>*0.017*		
Number of involved organs ≥3	2.19 (1.41-3.38)<br>*<0.001*	2.61 (1.4-4.85)<br>*0.002*		3.24 (1.16-9.08)<br>*0.025*
Number of involved soft tissue organs	1.51 (1.23-1.85)<br>*<0.001*	1.55 (1.16-2.07)<br>*0.003*		1.54 (1.02-2.33)<br>*0.042*
Pulmonary metastasis	1.98 (1.24-3.16)<br>*0.004*	2.15 (1.13-4.12)<br>*0.02*		3.24 (1.16-9.08)<br>*0.025*
Hepatic metastasis	1.51 (1.01-2.27)<br>*0.047*	1.84 (1.01-3.36)<br>*0.046*		
Lymphatic metastasis	1.6 (1.11-2.31)<br>*0.012*			2.46 (1.08-5.6)<br>*0.033*
Non-narcotics required	1.44 (1.0-2.07)<br>*0.049*		2.72 (1.08-6.87)<br>*0.034*	
Treatment-dependent parameters				
PSA response	0.44 (0.3-0.63)<br>*<0.001*	0.39 (0.24-0.65)<br>*<0.001*	0.27 (0.12-0.61)<br>*0.002*	
Pain response		0.57 (0.33-0.97)<br>*0.039*		
FT reduction = -100%		0.47 (0.23-0.96)<br>*0.039*		
FT nadir <0.2 pg/mL		0.29 (0.13-0.61)<br>*<0.001*		
FT nadir <0.18 pg/mL		0.32 (0.15-0.68)<br>*0.003*		
FT nadir <detection limit		0.32 (0.15-0.68)<br>*0.003*		
FT median <0.3 pg/mL		0.42 (0.21-0.82)<br>*0.011*		
FT median <0.2 pg/mL		0.38 (0.18-0.79)<br>*0.009*		
FT median <0.18 pg/mL		0.3 (0.12-0.75)<br>*0.01*		
FT median <detection limit		0.19 (0.06-0.57)<br>*0.003*		
TT median <detection limit	1.93 (1.23-3.05)<br>*0.005*			
**PSA response**				
Baseline parameters				
Previous prednisone		0.26 (0.08-0.81)<br>*0.02*		
LDH >ULN	2.73 (1.16-6.44)<br>*0.022*			
Progression of bone metastasis	2.28 (1.07-4.89)<br>*0.033*			

Narcotics required				7.33 (1.16-46.24) *0.034*
Treatment-dependent parameters				
Pain response	4.66 (2.16-10.07) *<0.001*	6.19 (1.9-20.17) *0.003*	5.44 (1.04-28.53) *0.045*	
Flare phenomenon			0.12 (0.02-0.63) *0.012*	
FT nadir <0.2 pg/mL		9.5 (1.69-53.33) *0.011*		
FT median <0.3 pg/mL		6.0 (1.47-24.55) *0.013*		
FT median <0.2 pg/mL	2.53 (1.05-6.1) *0.038*	10 (1.81-55.28) *0.008*		
FT median <0.18 pg/mL		9.85 (1.09-89.2) *0.042*		

Supplementary Table S1. Baseline and treatment-dependent parameters influencing progression-free survival, overall survival and PSA response: univariate analyses. *mDRPC* Metastatic docetaxel-resistant prostate cancer, *ART* androgen-receptor targeted therapy, *FT* free testosterone, *DL* detection limit, *CRP* c-reactive protein, *ULN* upper limit of normal, *LDH* lactate dehydrogenase, *NSE* neuron-specific enolase, *Hb* hemoglobin, *AP* alkaline phosphatase, *RECIST* Response Evaluation Criteria in Solid Tumors, *PSA* prostate-specific antigen, *ECOG* Eastern Cooperative Oncology Group, *TT* total testosterone.
